# Mitochondrial DNA alteration in obstructive sleep apnea

**DOI:** 10.1186/s12931-015-0205-7

**Published:** 2015-04-07

**Authors:** Donato Lacedonia, Giovanna E Carpagnano, Elisabetta Crisetti, Grazia Cotugno, Grazia P Palladino, Giulia Patricelli, Roberto Sabato, Maria P Foschino Barbaro

**Affiliations:** Department of Medical and Surgical Sciences, Institute of Respiratory Diseases, University of Foggia, Viale degli Aviatori, Foggia, 71100 Italy

**Keywords:** Mitochondrial DNA, OSAS, Oxidative stress, ROMs

## Abstract

**Background:**

Obstructive Sleep Apnea (OSAS) is a disease associated with the increase of cardiovascular risk and it is characterized by repeated episodes of Intermittent Hypoxia (IH) which inducing oxidative stress and systemic inflammation. Mitochondria are cell organelles involved in the respiratory that have their own DNA (MtDNA). The aim of this study was to investigate if the increase of oxidative stress in OSAS patients can induce also MtDNA alterations.

**Methods:**

46 OSAS patients (age 59.27 ± 11.38; BMI 30.84 ± 3.64; AHI 36.63 ± 24.18) were compared with 36 control subjects (age 54.42 ± 6.63; BMI 29.06 ± 4.7; AHI 3.8 ± 1.10). In blood cells Content of MtDNA and nuclear DNA (nDNA) was measured in OSAS patients by Real Time PCR. The ratio between MtDNA/nDNA was then calculated. Presence of oxidative stress was evaluated by levels of Reactive Oxygen Metabolites (ROMs), measured by diacron reactive oxygen metabolite test (d-ROM test).

**Results:**

MtDNA/nDNA was higher in patients with OSAS than in the control group (150.94 ± 49.14 vs 128.96 ± 45.8; p = 0.04), the levels of ROMs were also higher in OSAS subjects (329.71 ± 70.17 vs 226 ± 36.76; p = 0.04) and they were positively correlated with MtDNA/nDNA (R = 0.5, p < 0.01).

**Conclusions:**

In OSAS patients there is a Mitochondrial DNA damage induced by the increase of oxidative stress. Intermittent hypoxia seems to be the main mechanism which leads to this process.

## Introduction

Obstructive sleep apnea syndrome (OSAS) is a disease characterized by repetitive episodes of apnea and hypopnea during sleep, inducing cyclical alterations of arterial oxygen saturation/desaturation and sleep fragmentation. Intermittent hypoxia (IH) is the major pathophysiologic character of OSAS since it is the trigger of oxidative stress, systemic inflammation, and sympathetic activation. IH also causes the increase of reactive oxygen species (ROS) production [1] and increases the expression of inflammatory cytokines through activation of NF- κB [2,3].

Mitochondria are independent double membrane organelles found in the cytosol of eukaryotic cells which are involved in energy production, specifically they carry out oxidative phosphorylation (OXPHOS) [4]. Unlike the nuclear genome, mitochondriacontain unmethylated circular DNA and composed of one heavy strand and one light strand, organized into a nucleoprotein as a complex with the transcription factor A (TFAM) protein, that is responsible of the DNA packaging into compact nucleoids, which are found associated with the inner mitochondrial membrane [5]. The lack of introns, protective histones, and the close proximity to the electron transport chain result in mithocondrial DNA (MtDNA) being more susceptible to oxidative damage than nuclear DNA (nDNA). In addition to this, mitochondria have limited DNA repair capacity [6].

Our hypothesis is that the presence of oxidative stress can induce in OSAS patients an alteration of the transcriptional and replication machinery of mitochondrial biogenesis which would be up-regulated resulting in an increased mitochondrial biogenesis by replication of the mitochondrial genome. This change could be detected in body fluids. To test this hypothesis we used real time qPCR to measure mitochondrial to nuclear genome ratio (Mt/N) in accordance with the early theory which suggests that Mt/N is a biomarker of mitochondrial dysfunction [7].

## Materials and methods

### Population

Patients were consecutively enrolled in the Sleep Center of the University of Foggia from January 2012 to July 2013. The exclusion criteria were as follow: recent myocardial infarction or ictus, pregnancy, current smokers, patients with COPD, diabetes and tumors. (were also excluded). A spirometry (Sensormedics, USA) was performed to exclude chronic obstructive pulmonary disease (COPD), defined according to the GOLD guidelines as an irreversible chronic airway obstruction (FEV1/FVC ≤ 70%) in postbronchodilation spirometry. Patients with central apnoea and obesity hypoventilation syndrome were also excluded. The population was divided in two groups according to the poligraphy results: OSAS (AHI >5 events/hour) and control group (AHI ≤5). The protocol was approved by the Medical Ethics Committee at “Ospedali Riuniti of Foggia University” and all subjects provided a written informed consent before participating.

### Polygraphy

An unattended cardio-respiratory overnight monitoring (Vitalnight 11, Germany) was performed during the patient staying in the sleep laboratory. Sleep stages were not evaluated while flow, snoring, sleep position, SaO2 and heart rate were recorded. Oro-nasal flow was measured by a nasal canula, whereas abdominal and rib-cage movements were measured by pneumatic sensors and oxyhemoglobin saturation with a finger probe. Sleep-disordered breathing was quantified according to the standard criteria of the American Academy of Sleep Medicine manual [8]. The exam was considered to be good if there were at least 6 hours of registration. A manual score was performed the day after registration by a doctor specialized in sleep disorders.

### Blood collection and DNA extraction

A blood sample was obtained at wake up time the day after polygraphy. A total of 3 ml peripheral blood sample were collected in EDTA tubes and then was stored at −80°. Whole blood DNA was extracted with QIAamp DNA MiniKit according to the manufacturer’s protocol (Qiagen, Hilden, Germany). The concentration of extracted DNA was measured at 260 nm with NanoDrop Spectrophotometer (Thermo Scientific NanoDrop, USA) and was adjusted to 10 ng/μl. Extracted DNA was stored at −20° until further use.

### Quantitative real-time PCR

Mitochondrial DNA content was measured by quantitative real time PCR method using an Applied Biosystems 7300 real-time PCR System (PE Applied Biosystems). MtDNA was measured by quantification of a unique mitochondrial fragment relative to a single copy region of beta-2-microglobulin nuclear gene (β2M) [9]. Primers, Probes (IDT, Integrated DNA Technologies, USA) and gene accession numbers are listed in Table 1. Mitochondrial DNA and β2M probes were labelled at 5’ end with 6 FAM and MAX fluorescent dyes respectively and both probes contained BHQ-1 as a quencher dye at 3’ end. The PCR mix was: 1x TaqMan® Universal PCR Master Mix (PE Applied Biosystems), 200 nM of each primer, 125 nM of TaqMan Probe, 50 ng of total genomic DNA extract in a 20 μl PCR reaction. Quantitative real-time PCR conditions were 2 min at 50°C and 10 min at 95°C, followed by 40 cycles of 15 s of denaturation at 95°C and 60s of annealing/extension at 60°C. Standard curves obtained from serial dilutions of PCR-amplified target sequences were used for the quantification of MtDNA (Mt) and nuclear genome (N) then the ratio of Mt/N DNA was calculated.

**Table 1:** **Primers/probes used in the study**

**Gene accession number**	**Primer/probe**	**Sequence**	**Product size (bp)**
Human mithocondrial genome NC_012920	Mito F	TTAAACACATCTCTGCCAAACC	150
	Mito R	AGATTAGTAGTATGGGAGTGGGA	
	Mito P	AA CCC TAA CAC CAG CCT AAC CAG A	
Human β2M accession number M17987	β2M F	CTTTCTGGCTGGATTGGTATCT	100
	β2M R	CAGAATAGGCTGCTGTTCCTAC	
	β2M P	AG TAG GAA GGG CTT GTT CCT GCT G	

### The d-Roms test

Diacron reactive oxygen metabolite test (d-ROM test) was performed (Diacron SPF, Grosseto, Italy) to analyze the plasma levels of Reactive Oxygen Metabolites (ROMs). This test is based on the reaction of hydroperoxides of a biological sample with transition metals (iron) that catalyze the formation of free radicals which then oxidize an alkyilamine forming a colored radical detected by photometry at 505 nm. Ten μl of blood are mixed with 1 ml of an acidic (PH 4.8) buffer reagent (R2) in order to release iron from plasma proteins that will react with blood peroxides to form free radicals, and then 10 μl of a chromogen reagent (R1 reagent, alkyilamine) are added forming a pink-colored derivative. The concentration of these persistent species can be easily determined through spectrophotometric procedures (absorption at 505 nm). The results of the dROM test are expressed in arbitrary units called “Carratelli units” (U. CARR), where 1 U. CARR corresponds to 0.08 mg/dL of H_2_O_2_. Reference values of d-ROMs test are between 250 and 300 U. CARR. Values higher than 320 indicate increasing levels of oxidative stress.

### Blood gas analysis

Arterial blood sample for the analysis of gases during room-air breathing was drawn with the patient in sitting position, the day after polygraphy registration and within one hour of waking up. PaO2, PaCO2 and pH were measured in a blood gas analyzer (Model 1312; Instrumentation Laboratory; Milan, Italy).

### Statistical analysis

Descriptive statistics (i.e., means, standard deviations, percentages) were applied to summarize the continuous and categorical variables. The relationship between two continuous variables was determined by measuring the Pearson’s correlation coefficient. All variables analyzed were normally distributed so Student’s *T*-test was used to compare the mean values. Multiple regression analysis was also used to evaluate the influence of different factors on MtDNA level. P value < 0,05 was considered significant. Statistical Software (Statistica version 8.0, StatSoft, Inc. 2007, USA) was used to analyze the data.

## Results

Among patients potentially eligible, 82 patients were enrolled according to the inclusion criteria and divided in 46 OSAS and 36 controls respectively. Table 2 shows general characteristics of populations. Between control subjects and OSAS group there are no differences of age and BMI. The groups were also similar for gas exchange and pulmonary function.

**Table 2:** **General characteristics of population and results of mitochondrial and nucluear DNA analysis**

	**Patients (N = 46)**	**Controls (N = 36)**	
	***Mean*** **±** ***DS***	***Mean*** **±** ***DS***	***p***
*General Characteristics*			
Males (%)	*76*	73	0.67
Age (years)	59.27 ± 11.38	54.42 ± 6.63	0.08
BMI (Kg/m^2^)	30.84 ± 3.64	29.06 ± 4.7	0.07
pH	7.40 ± 0.02	7.40 ± 0.01	0.60
PaO2 (mmHg)	80.78 ± 11.65	71.90 ± 18.00	0.13
PaCO2 (mmHg)	40.95 ± 4.45	40.42 ± 5.51	0.80
FVC (%)	104.89 ± 18.55	103.25 ± 14.05	0.37
FEV_1_ (%)	97.60 ± 25.02	96.58 ± 11.65	0.85
FEV_1_/FVC	75.37 ± 4.2	78.2 ± 6.1	0.30
Polygraphic Data			
AHI (events/h)	36.63 ± 24.18	3.8 ± 1.10	<0,001
ODI (events /h)	28.51 ± 25.31	3,1 ± 1.00	<0,001
T90 (%)	16.98 ± 22.98	1,5 ± 0.31	<0,001
SaO2 Mean (%)	91.51 ± 5.65	95,1 ± 2.42	<0,001
ESS	11.35 ± 3.68	4.3 ± 2.15	< 0,001
Biological Data			
MtDNA/nDNA	150.94 ± 49.14	128.96 ± 45.80	0.04
Ct MtDNA/nDNA	0.67 ± 0.02	0.69 ± 0.03	0.01
Log MtDNA	2.16 ± 0.14	2.08 ± 0.19	0.04
ROMs	329.71 ± 70.17	226.00 ± 36.76	0.04

*Abbreviations: BMI* Body Mass Index, *AHI* Apnea Hypopnea Index, *ODI* Oxygen Desaturation Index, *T90* Total time with SaO2 under 90%, *ESS* Epworth Sleepiness Scale.

**Figure 1:**
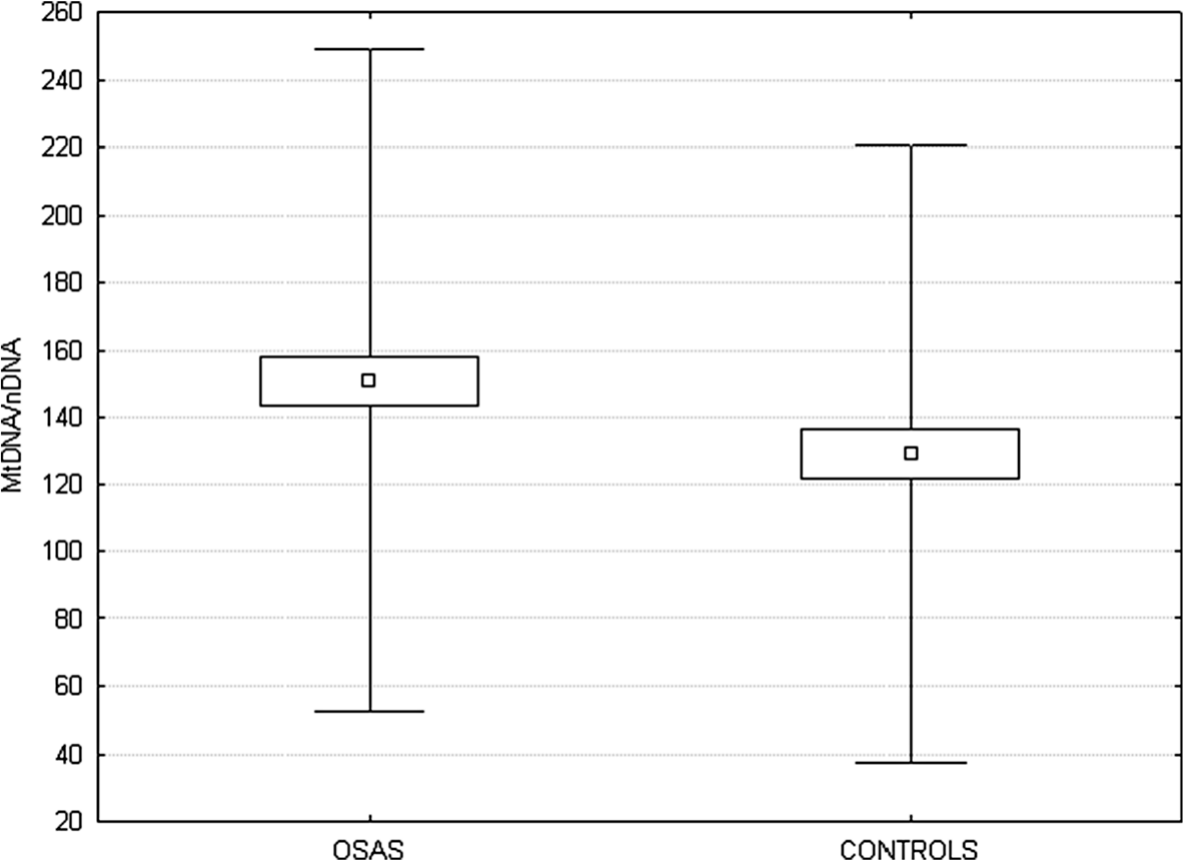
**Differences between ratio mitochondrial/nuclear DNA in OSAS subjects and in control group.** (□ median with range, CI 25-75%, p = 0.04).

**Figure 2:**
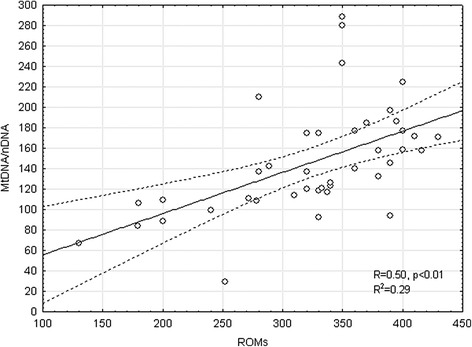
**Correlation between ratio mitochondrial/nuclear DNA and level of ROMs in OSAS patients: R = 0.5, p < 0.01.** (Dashed lines rappresent CI 95%).

OSAS patients showed higher level of Mitochondrial DNA/nuclear DNA ratio (150.94 ± 49.14 vs 128.96 ± 45.8; p = 0.04) and lower Ct (Threshold Cycle) (0.67 ± 0.02 vs 0.69 ± 0.03; p = 0.01) than the control group (Figure 1). The level of ROMs was also higher in OSAS subjects (329.71 ± 70.17 vs 226 ± 36.76; p = 0.04). The level of MtDNA/nDNA was correlated with diurnal PaO2, FEV1% and ROMs (Table 3, Figure 2). Age, body mass index (BMI) and the main sleep parameters (AHI, ODI and T90) did not influence directly mitochondrial DNA. Multiple regression analysis including PaO2, FEV1 and ROMs shows that only the levels of ROMs were correlated with MtDNA/nDNA (R = 0.71; Beta = 0.67; Stand. Err. = 0.15; t = 4.2; p < 0.01).

**Table 3:** **Correlation between each variable (R)**

	***PaO2***	***MtDNA/nDNA***	***ROMs***
*BMI*	**−0.37**	0.23	0.13
*PaO2*		**−0.32**	−0.19
FEV_1_%	0.24	**−0.30**	−0.02
*AHI*	**−0.38**	0.007	−0.20
*T90n*	**−0.59**	0.11	0.14
*ODI*	**−0.46**	0.13	0.005
*ROMs*		**0.5**	

In bold the results which were significant (p < 0.05) in OSAS.

## Discussion

The main finding of our study is that OSAS patients have an alteration of MtDNA content and this seems to be related with the increase of oxidative stress levels. OSAS is a common condition characterized by intermittent hypoxia (IH). IH acts as a trigger of oxidative stress, systemic inflammation, and sympathetic activation. In turn, an increased oxidative stress will lead to activation of nuclear factor (NF)-κB, and hence increased expression of a number of downstream NF-κB target genes, for example proinflammatory cytokines (TNF-α, IL-6, and IL-8) as well as intercellular adhesion molecules and others basic biomarkers of inflammatory status such as C-Reactive Proteine (CRP) or Fibrinogen [10-12]. On the other hand in vitro studies it was well documented [13] that IH can also induce mitochondrial alterations and MtDNA injuries. So, we can speculate also that the presence of ischemia-reperfusion injury in sleep apnoea could induce a mitochondrial damage and an alteration of MtDNA.

The human mitochondrial genome is particularly susceptible to alterations because mithocondria have few repair mechanisms so, an increased oxidative stress could be very deleterious to it [14]. Different methods to study MtDNA are available, however recent studies suggest that the measure of content of MtDNA estimated by the mitochondrial to nuclear genome ratio (Mt/N) is a simple way to evaluate the presence of mitochondrial dysfunctions. The number of mitochondria in a particular cell type can vary depending on many factors, including the stage of cell cycle, environment and redox balance of the cell, stage of differentiation, and a different cell signaling mechanisms [15]. Individual mitochondria can contain several copies of the mitochondrial genome [16]. A decrease in the number of MtDNA copies has been associated with renal cell carcinoma and breast cancer while, by contrast, an increased MtDNA copy number was observed in esophageal squamous cell carcinoma and other chronic inflammatory diseases [17].

The presence of oxidative stress seems to be the main cause of change in MtDNA copy number. Mitochondria are the major site of reactive oxygen species (ROS) generation, produced during the ATP production by electron leakage that occurs in the mitochondrial electron transport chain [18]. As well as in energy production, mitochondria are also involved in the regulation of numerous other cellular functions including cell proliferation, apoptosis, and intracellular calcium homeostasis [7]. Several studies have shown how these disorders are related to a condition of oxidative stress [7].

Oxidative stress is the result of a disorder of the redox balance of the cell, resulting in excessive oxidation of intracellular proteins. Oxidation and reduction of proteins are a major signalling mechanism of intracellular control and are usually mediated via sulfhydril groups of cysteines in proteins and can affect almost all cellular processes including protein folding, protein activity, and numerous biochemical pathways [19]. Therefore, an alteration of cells redox balance can have major implications on cell signaling, resulting in cell alterations that could impair the normal function and lead to disease [20].

It is known that chronic oxidative stress can cause damage to proteins, lipids and DNA molecules within the cell and it is considered to play a role in many common diseases such as diabetes and its complications, cancer [21], neurodegenerative disorders [22] and OSAS [23].

In the same way, oxidative stress induces mitochondrial dysfunctions even if its molecular mechanisms are not well understood. Multiple pathways may converge in mitochondria to modify respiratory chain activity. Moreover, recent studies indicate that the production of ROS may be accompanied by changes in mitochondrial metabolism [24]. In these damaged mitochondria the electron transport chain may be blocked, resulting in accumulation of excess of free radicals [25]. As mitochondrial DNA is located close to the source of ROS production, the DNA itself can become damaged resulting in an accumulation of deletions and mutations [26]. An accumulation of such damaged MtDNA in the cell may result in a chronic innate inflammatory response.

We know that reactive oxidative stress (ROS) is increased in OSAS [27] and cyclic changes in arterial oxygen saturation are the main responsible of ROS production [28]. During ischemia, ROS production starts to increase and after few minute of reperfusion there is a peak of oxidative stress [29].

Mitochondrial components and mitochondrial membrane are highly susceptible to be attacked by reactive oxygen species (ROS) [30], in fact in conditions of oxidative stress there is the opening of mitochondrial permeability transition pore, cytochrome C releasing, and mitochondrial apoptosis [31]. In case of acute IH the endogenous anti-oxidant molecules can contrast deleterious effects of ROS, but in OSAS patients the presence of a persistent IH maintains high level of ROS in the cell which leads, to mitochondria dysfunction and MtDNA alteration and others dangerous effects.

Sleep fragmentation could be another possible mechanism that can induce an alteration of mitochondrial function. In fact, it is well known that sleep deprivation can lead to an imbalance between sympathetic and parasympathetic system with many consequences, in particular on cardiovascular system (higher blood pressure levels, increased risk of arrhythmia and stroke) and on some metabolic diseases such as obesity and diabetes. It was demonstrated that sleep fragmentation can induce a pro-inflammatory status with an increase of IL-6 and TNF-alfa and it also activates an adaptive stress pathway which has deleterious consequence of reactive oxygen species [32]. Thus, we can suppose that sleep deprivation can induce mitochondrial damage by the same mechanism.

Because mitochondria are involved in several fundamental cellular processes, their dysfunction can affect a range of important cellular functions and can lead to a variety of diseases [15]. The role of mitochondrial dysfunction in numerous diseases is well documented [9]. Studies looking specifically at alterations in MtDNA content in various cell types cover a broad range of human diseases, such as diabetes and its complications [33], obesity [34], cancer [35-38], cardiovascular diseases [39], COPD [40] and others. So, the presence of mitochondrial dysfunctions in OSAS subjects could be not only a simple consequence of IH but also one of the mechanisms which contribute to increase other diseases frequently associated with OSAS such as cardiovascular, metabolic and inflammatory diseases.

### Study limitations

There are some limitations in this study. First, a complete Polysomongraphy was not performed so we have no information about sleep fragmentation and how it could have influenced the oxidative stress and mtDNA alterations. Second, there is not a clear consensus about this method because any standard values of Mt/N are available up today. Finally, MtDNA content changes in different cells types, so tissues MtDNA could be differently influenced by IH compared to blood cells. Thus, even if the results of present study are encouraging, it is early to affirm with certainty that there is a direct link between OSAS and MtDNA damage. In fact the limited number of subjects involved in the study and the relative low level of correlation coefficient among all variables considered (almost 0.5) although statically significant maybe are not enough to give a definitive answer about our hypothesis. Others variables such as obesity, drugs, years of OSAS history could be involved and needs to be excluded in the further study. Anyway we would underline that this is a pilot study in this field and our hypothesis should be confirmed by others studies.

## Conclusion

Our results suggest that in OSAS subjects the transcriptional and replication machinery of mitochondrial biogenesis will be up-regulated resulting in an increased mitochondrial biogenesis via replication of the mitochondrial genome and this change could be detected in body fluids. The conditions of oxidative stress due to repetitive episodes of ischemia-reperfusion are likely the main way to induce these dysfunctions. From our point of view this research field could open interesting sceneries to better understand the pathophysiology of diseases linked to OSAS as well as in others respiratory diseases in which oxidative stress is always involved such as COPD or pulmonary interstitial lung disease.

## References

[1] Lavie L (2003). Obstructive sleep apnoea syndrome – an oxidative stress disorder. Sleep Med Rev.

[2] Hücking K, Hamilton-Wessler M, Ellmerer M, Bergman RN (2003). Burst-like control of lipolysis by the sympathetic nervous system in vivo. J Clin Invest.

[3] Nguyen MT, Satoh H, Favelyukis S, Babendure JL, Imamura T, Sbodio JI (2005). JNK and tumor necrosis factor-alpha mediate free fatty acid-induced insulin resistance in 3 T3-L1 adipocytes. J Biol Chem.

[4] Di Donato S, Marmolino D, Taroni F (2013). Mitochondrial disorders handbook of the cerebellum and cerebellar disorders.

[5] Bogenhagen DF (2012). Mitochondrial DNA nucleoid structure. Biochim Biophys Acta.

[6] Lee HC, Lu CY, Fahn HJ, Wei YH (1998). Aging- and smoking-associated alteration in the relative content of mitochondrial DNA in human lung. FEBS Lett.

[7] Malik AN, Czajka A (2013). Is mitochondrial DNA content a potential biomarker of mitochondrial dysfunction?. Mitochondrion.

[8] American Academy of Sleep Medicine (2005). The International Classification of Sleep Disorders.

[9] Malik AN, Shahni R, Rodriguez-de-Ledesma A, Laftah A, Cunningham P (2011). Mitochondrial DNA as a non-invasive biomarker: accurate quantification using real time quantitative PCR without co-amplification of pseudogenes and dilution bias. Biochem Biophys Res Commun.

[10] Blasi A, Jo JA, Valladares E, Juarez R, Baydur A, Khoo MC (2006). Autonomic cardiovascular control following transient arousal from sleep: a time-varying closed-loop model. IEEE Trans Biomed Eng.

[11] Meier-Ewert HK, Ridker PM, Rifai N, Regan MM, Price NJ, Dinges DF (2004). Effect of sleep loss on C-reactive protein, an inflammatory marker of cardiovascular risk. J Am Coll Cardiol.

[12] Irwin MR, Wang M, Campomayor CO, Collado-Hidalgo A, Cole S (2006). Sleep deprivation and activation of morning levels of cellular and genomic markers of inflammation. Arch Intern Med.

[13] Loor G, Kondapalli J, Iwase H, Chandel NS, Waypa GB, Guzy RD (2011). Mitochondrial oxidant stress triggers cell death in simulated ischemia-reperfusion. Biochim Biophys Acta.

[14] Chatterjee A, Mambo E, Sidransky D (2006). Mitochondrial DNA mutations in human cancer. Oncogene.

[15] Michel S, Wanet A, De Pauw A, Rommelaere G, Arnould T, Renard P (2012). Crosstalk between mitochondrial (dys)function and mitochondrial abundance. J Cell Physiol.

[16] Navratil M, Poe BG, Arriaga EA (2007). Quantitation of DNA copy number in individual mitochondrial particles by capillary electrophoresis. Anal Chem.

[17] Clay Montier LL, Deng JJ, Bai Y (2009). Number matters: control of mammalian mitochondrial DNA copy number. J Genet Genomics.

[18] Bonner MR, Shen M, Liu CS, Divita M, He X, Lan Q (2009). Mitochondrial DNA content and lung cancer risk in Xuan Wei. China Lung Cancer.

[19] Hurd TR, Costa NJ, Dahm CC, Beer SM, Brown SE, Filipovska A (2005). Glutathionylation of mitochondrial proteins. Antioxid Redox Signal.

[20] Lee J, Giordano S, Zhang J (2012). Autophagy, mitochondria and oxidative stress: cross-talk and redox signalling. Biochem J.

[21] Dasgupta S, Soudry E, Mukhopadhyay N, Shao C, Yee J, Lam S (2012). Mitochondrial DNA mutations in respiratory complex-I in never-smoker lung cancer patients contribute to lung cancer progression and associated with EGFR gene mutation. J Cell Physiol.

[22] Sas K, Robotka H, Toldi J, Vécsei L (2007). Mitochondria, metabolic disturbances, oxidative stress and the kynurenine system, with focus on neurodegenerative disorders. J Neurol Sci.

[23] Kang IG, Jung JH, Kim ST (2013). The effect of obstructive sleep apnea on DNA damage and oxidative stress. Clin Exp Otorhinolaryngol.

[24] Pelicano H, Lu W, Zhou Y, Zhang W, Chen Z, Hu Y (2009). Mitochondrial dysfunction and reactive oxygen species imbalance promote breast cancer cell motility through a CXCL14-mediated mechanism. Cancer Res.

[25] Giacco F, Brownlee M (2010). Oxidative stress and diabetic complications. Circ Res.

[26] Indo HP, Davidson M, Yen HC, Suenaga S, Tomita K, Nishii T (2007). Evidence of ROS generation by mitochondria in cells with impaired electron transport chain and mitochondrial DNA damage. Mitochondrion.

[27] Carpagnano GE, Kharitonov SA, Resta O, Foschino-Barbaro MP, Gramiccioni E, Barnes PJ (2003). 8-Isoprostane, a marker of oxidative stress, is increased in exhaled breath condensate of patients with obstructive sleep apnea after night and is reduced by continuous positive airway pressure therapy. Chest.

[28] Christou K, Markoulis N, Moulas AN, Pastaka C, Gourgoulianis KI (2003). Reactive oxygen metabolites (ROMs) as an index of oxidative stress in obstructive sleep apnea patients. Sleep Breath.

[29] Ambrosio G, Zweier JL, Duilio C, Kuppusamy P, Santoro G, Elia PP (1993). Evidence that mitochondrial respiration is a source of potentially toxic oxygen free radicals in intact rabbit hearts subjected to ischemia and reflow. J Biol Chem.

[30] Wallace DC, Fan W (2010). Energetics, epigenetics, mitochondrial genetics. Mitochondrion.

[31] Di Lisa F, Canton M, Menabò R, Dodoni G, Bernardi P (2003). Mitochondria and reperfusion injury. The role of permeability transition. Basic Res Cardiol.

[32] Levy P, Tamisier R, Arnaud C, Monneret D, Baguet JP, Stanke-Labesque F (2012). Sleep deprivation, sleep apnea and cardiovascular diseases. Front Biosci.

[33] Malik AN, Shahni R, Iqbal MM (2009). Increased peripheral blood mitochondrial DNA in type 2 diabetic patients with nephropathy. Diabetes Res Clin Pract.

[34] Kaaman M, Sparks LM, van Harmelen V, Smith SR, Sjölin E, Dahlman I (2007). Strong association between mitochondrial DNA copy number and lipogenesis in human white adipose tissue. Diabetologia.

[35] Ma J, Zhang Q, Chen S, Fang B, Yang Q, Chen C (2013). Mitochondrial dysfunction promotes breast cancer cell migration and invasion through HIF1α accumulation via increased production of reactive oxygen species. PLoS One.

[36] Yang Ai SS, Hsu K, Herbert C, Cheng Z, Hunt J, Lewis CR (2013). Mitochondrial DNA mutations in exhaled breath condensate of patients with lung cancer. Respir Med.

[37] Wang Y, Liu VW, Xue WC, Tsang PC, Cheung AN, Ngan HY (2005). The increase of mitochondrial DNA content in endometrial adenocarcinoma cells: a quantitative study using laser-captured microdissected tissues. Gynecol Oncol.

[38] Xing J, Chen M, Wood CG, Lin J, Spitz MR, Ma J (2008). Mitochondrial DNA content: its genetic heritability and association with renal cell carcinoma. J Natl Cancer Inst.

[39] Marzetti E, Csiszar A, Dutta D, Balagopal G, Calvani R, Leeuwenburgh C (2013). Role of mitochondrial dysfunction and altered autophagy in cardiovascular aging and disease: from mechanisms to therapeutics. Am J Physiol Heart Circ Physiol.

[40] Slebos DJ, van der Toorn M, Bakker SJ, Kauffman HF (2007). Mitochondrial dysfunction in COPD patients with low body mass index. Eur Respir J.

